# Identification of targets for introgression from rye (*Secale* ssp.) into wheat (*Triticum aestivum*) to increase arabinoxylan content in flour using a genome-wide association study

**DOI:** 10.3389/fpls.2026.1892461

**Published:** 2026-07-22

**Authors:** Maria Chiara Piro, Tom Ruttink, Hilde Muylle, Steven Maenhout, Geert Haesaert

**Affiliations:** 1 Department of Plants and Crops, Faculty of Bioscience Engineering, Ghent University, Ghent, Belgium; 2 Plant Sciences Unit, Flanders Research Institute for Agriculture, Fisheries and Food (ILVO), Melle, Belgium; 3 Department of Plant Biotechnology and Bioinformatics, Faculty of Sciences, Ghent University, Ghent, Belgium

**Keywords:** arabinoxylan, grain quality, GWAS, wheat-alien introgression, rye

## Abstract

Among cereal species, rye (*Secale cereale* L.) has the highest dietary fibre content, particularly arabinoxylans (AX). Thus, rye flour has gained popularity as a bioactive supplement to white wheat flour-based products. An alternative approach to bio-fortifying white wheat flour could be through wheat-rye introgressions. The recent availability of a rye reference genome assembly makes genome-wide association studies (GWAS) possible for this allogamous species, simplifying the identification of AX-associated rye introgression targets. In this work, a collection comprising 317 rye accessions was assembled to investigate the genetic basis of rye grain quality traits, including total AX (TOT-AX), water-extractable AX (WE-AX) and water-unextractable AX (WU-AX) contents, the proportion of WE-AX to TOT-AX, and thousand kernel weight (TKW). The collection was genotyped using a genotyping-by-sequencing (GBS) approach. Genetic diversification based on the regionally predominant end use of rye was phenotypically evident when considering the TKW of rye. The GWAS detected 12 marker-trait associations (MTAs). No MTA for TKW colocalised with an MTA for TOT-AX, WE-AX, or WU-AX contents. Two MTAs for WE-AX and WU-AX contents located on chromosome segment 2RL explained 21–26% of phenotypic variance, and two MTAs for TOT-AX and WU-AX contents colocalised on chromosome segment 7RL explained about 33% of phenotypic variance. Despite the lack of candidate genes with known cell wall-related functions within LD range of these MTAs, these associations deserve further attention as introgression targets for AX-targeted breeding in wheat.

## 1 Introduction

Rye flour (*Secale cereale* L.) is, second only to wheat flour (*Triticum aestivum* L.), the most common ingredient in bread and other baked products (Deleu et al., 2020; Kamal-Eldin et al., 2008). Rye flour of different extraction rates finds different applications in the baking industry. Light flours with an extraction rate in the range of 72–80% are extensively applied in the baking industry, and rye flour can often be found in blends with wheat flour (Capuano et al., 2010; Cyran and Ceglinska, 2011; Kamal-Eldin et al., 2008). Wheat-rye blends have gained popularity in the food industry, owing to the higher dietary fibre (DF) content of rye flour (Gao et al., 2018; Meeus et al., 2020). DF is associated with several health benefits, from the regulation of postprandial blood glucose and insulin levels to the modulation of the composition of the colonic microbiome (Garcia et al., 2007; Neyrinck et al., 2021). The DF content in rye flour ranges between 5.5–10% of dry matter (dm), whereas the DF content in wheat flour ranges between 2.7–4.7% dm (Andersson et al., 1993; Gebruers et al., 2008; Nilsson et al., 1997; Nyström et al., 2008). Thus, rye flour is considered a health-promoting ingredient, and it is promoted as a bioactive supplement to white wheat flour-based products.

The making of wheat-rye blends is not the only way to increase the DF provision of white wheat flour-based products. Another strategy that has recently gained popularity is targeting DF components such as arabinoxylan (AX) through selection (Shewry et al., 2024; Tremmel-Bede et al., 2017, 2020). AX is a hemicellulose and the main cell wall component in rye and wheat (Cyran and Saulnier, 2005; Gartaula et al., 2018). AX accounts for about half of the total DF in rye and wheat flours. Currently, the maximum reported total AX (TOT-AX) content in white wheat flour of wheat breeding lines selected for an increased AX content is 2.37% dm (Tremmel-Bede et al., 2017), whereas the AX content in rye flour can range between 3.11–4.31% dm (Glitsø and Bach Knudsen, 1999; Hansen et al., 2003; Nyström et al., 2008). Thus, the introgression of rye chromosome segments in wheat holds potential for further increasing the AX content in wheat flour. Rye introgressions are a well-established practice in the wheat breeding sector that has been mainly exploited for the improvement of disease resistance (Crespo-Herrera et al., 2017; Lukaszewski, 2015). Nevertheless, effects of such introgressions on a variety of quality traits, including DF and AX content, have already been described in the literature (Johansson et al., 2024).

The general structure of rye and wheat AX is essentially equivalent. AX features a backbone of (1-4)-linked β-D-xylopyranosyl (Xyl*p*) residues. The Xyl*p* residues can be monosubstituted by α-L-arabinofuranosyl (Ara*f*) residues on the O-2 or O-3 positions, or they can be di-substituted on both positions simultaneously (Saulnier et al., 2007). The O-3-linked Ara*f* residues can be decorated on the O-5 position by hydroxycinnamic acids, primarily *trans-*ferulic acid. Adjacent AX chains can be joined together through the oxidative cross-link between ferulic acid residues present on both chains, yielding diferulate bridges (Saulnier et al., 2007). The degree of diferulate cross-linking and the degree of substitution of the Xyl*p* residues with Ara*f* residues affect the solubility of AX. Thus, AX is generally distinguished into two fractions: water-extractable AX (WE-AX) and water-unextractable AX (WU-AX; Saulnier et al., 2007). Rye AX is characterised by a higher Ara*f* to Xyl*p* ratio compared to wheat AX (i.e., 0.66–0.76 for rye AX, and 0.50–0.70 for wheat AX; Gebruers et al., 2008; Nyström et al., 2008), and by a higher average molecular weight (i.e., 17.0 × 10^5^–17.4 × 10^5^ g/mol for rye AX, and 11.4 × 10^5^–12.2 × 10^5^ g/mol for wheat AX; Rakha et al., 2011).

Few studies have focussed on the genetic aspects of AX content in rye. Previous studies attempting to partition the phenotypic variance of AX content in rye in its genetic and environmental components have yielded contradictory results. Hansen et al. (2003) reported that both genotype and harvest year had a significant effect on TOT-AX content in rye wholemeal, whereas the variation in WU-AX content was mainly determined by the effect of the harvest year, and neither genotype nor harvest year had a significant effect on WE-AX content (Hansen et al., 2003). Shewry et al. (2010), determined the effects of genotype and environment (i.e., combination of harvest year and trial location) on the AX content in rye flour and bran. The environment had a significant effect on the TOT-AX and WE-AX contents in rye flour and bran, whereas the effect of the genotype was only significant with respect to the TOT-AX content in rye bran (Shewry et al., 2010). To our knowledge, the work of Siekmann et al. (2021), offers the only estimation of broad sense heritability (*H^2^*) of AX related traits in rye. They estimated *H^2^* of TOT-AX content in rye wholemeal to be 89% and that of WE-AX content in wholemeal to be 75%, which would suggest a considerable genotypic effect on both traits.

The characterisation of wheat-rye translocation and addition lines has played a significant role in the determination of genomic regions controlling AX content in rye. Using this plant material, it was shown that the introgression of chromosomes 1R, 2R, 4R, 5R, and 6R could improve the AX content in wheat (Boros et al., 2002; Schneider et al., 2016; Szakács et al., 2016, 2020). However, the vast majority of wheat cultivars currently available that are carriers of a rye introgression carry either the 1BL.1RS or 1AL.1RS translocations or the 1B(1R) substitution (Schlegel, 2023). The introgression of the 1RS segment is notoriously detrimental to gluten and baking quality because of the concomitant introgression of the rye *Sec-1* locus, which encodes 40K γ-secalin and ω-secalin storage proteins (Shewry et al., 1986; Graybosch et al., 1993). Therefore, breeders interested in exploiting rye as a genetic resource for AX-targeted breeding in wheat need novel AX-associated rye introgression targets that do not interfere with other key grain quality traits of wheat.

Since whole-genome reference assemblies have become available for rye (Li et al., 2021; Rabanus-Wallace et al., 2021), these new genomic resources have been exploited in transcriptomic analyses, genome-wide association studies (GWAS), and the identification of wheat orthologous genes in rye (Båga et al., 2022; Kozlova et al., 2022; Krępski et al., 2023; Siekmann et al., 2021; Sun et al., 2022; Vendelbo et al., 2021, 2022). Inbred lines from hybrid rye breeding programmes have been the plant material of choice in GWAS (Siekmann et al., 2021; Vendelbo et al., 2021, 2022). Such lines belong to single heterotic pools, such as the “Petkus” and “Carstens” gene pools used by Siekmann et al. (2021)—the two main heterotic pools used in rye breeding in Central Europe (Fischer et al., 2010)—or the “Gülzow” gene pool from Denmark used by Vendelbo et al. (2021, 2022). Nevertheless, cultivated rye is a crop with a long and complex domestication history, and geographically discrete genetic clusters of rye landraces have been described (Hagenblad et al., 2016). Thus, it is interesting to include landraces with a broad geographical distribution in GWAS to access genetic diversity not yet exploited in mainstream breeding programmes. Including landraces of an allogamous species such as rye implies that genotyping is performed at population level, rather than individuals. This can be achieved through genotyping-by-sequencing (GBS) of pools of individuals, which has proved effective when working with allogamous species, including rye (Båga et al., 2022; Knorst et al., 2019; Pégard et al., 2023; Zanotto et al., 2023). Marker-trait associations (MTAs) identified by GWAS constitute amenable targets for the development of molecular markers that can be used for the quick screening of donor material as well as of early generation wheat-rye introgression lines. By performing a GWAS on a diverse collection of rye landraces, the present study aims to uncover MTAs related to the TOT-AX, WE-AX, and WU-AX contents in rye flour, and to other key grain quality traits such as TKW. The resulting MTAs enable breeders to optimise wheat-rye introgression strategies and support targeted selection for AX content.

## 2 Materials and methods

### 2.1 Plant material and growing conditions

The *Secale* collection used in the present study comprised 312 rye accessions (*Secale cereale* subsp., *S. strictum* subsp., *S. sylvestre*, *S. vavilovii*), originating from 43 countries across seven geographical regions of origin (i.e., Northern Europe, Southern Europe, Central Europe, Western Europe, Eastern Europe, West and Central Asia, Other; **Supplementary Table 1**). The seed material was provided by the Genebank of Leibniz-Institute of Plant Genetics and Crop Plant Research, IPK (Gatersleben, Germany), and by the National Small Grains Germplasm Research Facility, USDA-ARS (Aberdeen, WA, United States). Five cultivars were added to the collection as check varieties, including three hybrid varieties (i.e., “Helltop”, “Poseidon”, “Stannos”) and two open-pollinated varieties (i.e., “KWS Bono”, “Duiker Max”).

Two field trials, hereafter referred to as S20/H21 and S21/H22, were conducted at the HOGENT/UGent Bottelare Experimental Farm (latitude 50°57’43”, longitude 3°45’37’’) in the growing seasons of 2020–2021 and 2021-2022, respectively. Both field trials were laid out in an augmented design with five replicated check varieties. Each accession and check variety was sown in a micro-plot of 1 m², comprising six rows spaced 20 cm from one another. The two central rows of the microplot were sown with 48 seeds of the rye accession or check variety, whereas the two flanking rows on each side were sown with a winter wheat variety of local use, “Mentor”. The microplots were arranged in 20 rows and 18 columns. The field was divided in two super-rows, defined as a blocking unit of 10 adjacent rows, three super-columns, defined as a blocking unit of six adjacent columns, and six blocks, defined as the intersection between super-rows and super-columns. Each check variety was present in each block, for a total of six replications per check variety. Mineral fertilisation was adjusted based on the results of the soil analysis performed by the Soil Service of Belgium (Heverlee, Belgium). Pest and disease management was adjusted based on the season’s pest and disease pressure and followed local agronomic practices.

**Figure 1:**
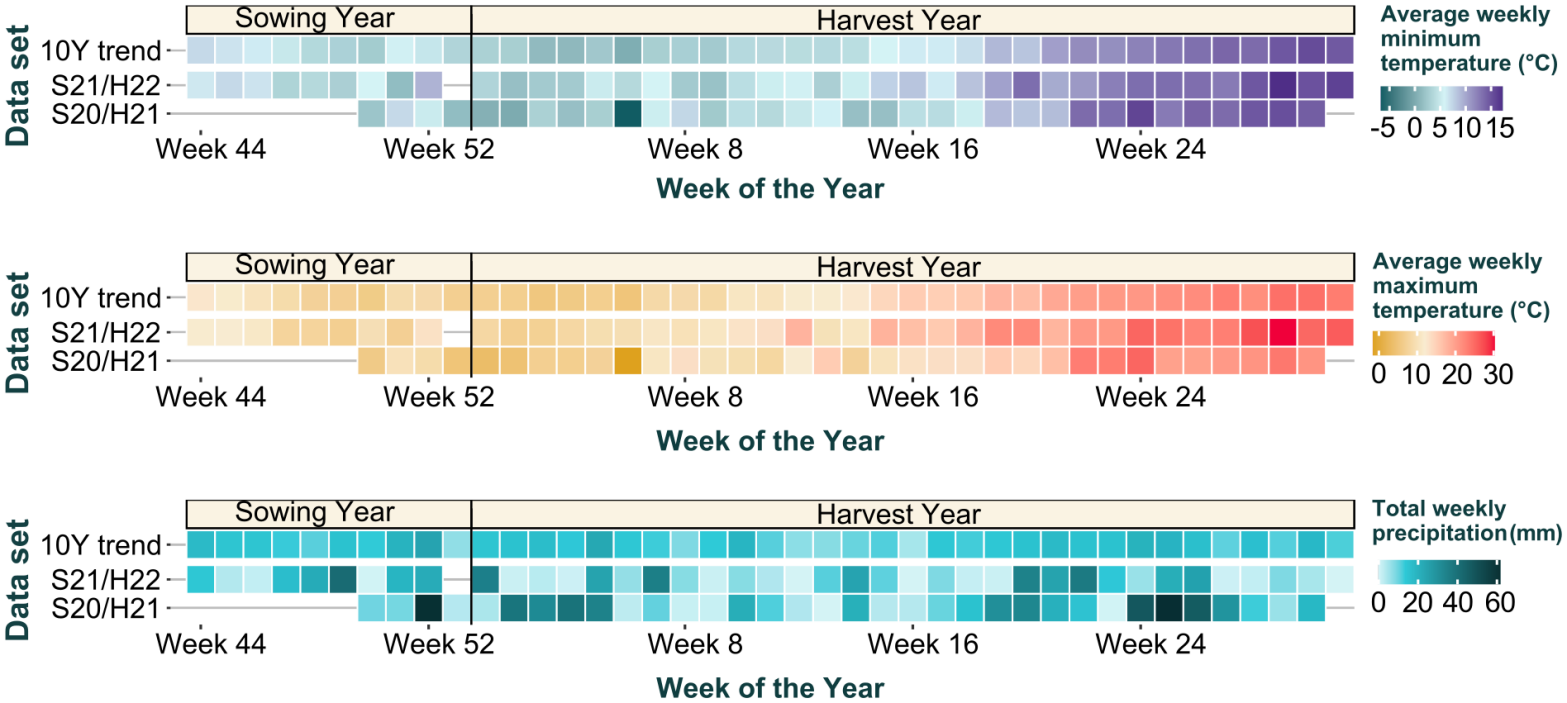
Tile plots of weather parameters including average weekly minimum temperature (°C), average weekly maximum temperature (°C), and total weekly precipitation (mm). Data are aggregated by week following the ISO 8601 convention. Data displayed include the local weather trend over the 10-year period 2012-2022 (10Y trend), and the weather trends registered during the two trials (S20/H21: trial of season 2020-2021, S21/H22: trial of season 2021-2022). Measurements of the weather parameters during each trial start at the week of sowing and end at the week of harvest.

Due to adverse weather conditions, trial S20/H21 could be sown only on 08/12/2020 and lasted 34 weeks in total. Trial S21/H22was sown on 02/11/2021 and lasted 40 weeks in total. Flowering commenced on 02/06/2021 during trial S20/H21 and on 10/05/2022 during trial S21/H22. Weather data were retrieved from the local weather station, and included daily minimum temperature (°C), daily maximum temperature (°C), and daily total precipitation (mm). Considering the overlapping trial weeks, trials S20/H21 and S21/H22 experienced comparable trends of average minimum temperature and average maximum temperature. Trial S20/H21 was characterised by a higher total precipitation as compared to trial S21/H22. In particular, the weeks between flowering and harvest of trial S20/H21 received 236 mm of cumulative precipitation, whereas the same period of trial S21/H22 received 178 mm of cumulative precipitation (**Figure 1**).

### 2.2 Phenotyping of rye kernels

Thousand kernel weight (TKW) was determined by weighing two independent samples of 500 rye kernels, counted with a CONTADOR seed counter (PAWLICA^®^, Drnovská, Czech Republic). Grain moisture was determined using a DICKEY-john GAC500XT moisture tester (DICKEY-John^®^, Auburn, IL, USA). The measured TKW was normalised to 15% grain moisture (gm).

A total of 100 g of rye kernels was conditioned to 13.5% gm and then milled with a Sedimat laboratory mill (Brabender^®^, Duisburg, Germany), which is equipped with a 150 µm mesh sifter, to obtain endosperm-enriched flour. The TOT-AX and WE-AX contents in flour were determined by a phloroglucinol colorimetric assay. Each sample set consisted of 15 different rye flour samples in single technical replicate, and one reference flour sample of the wheat variety “Evina” in triplicate, for a total of 18 samples per sample set. Analysis of TOT-AX and WE-AX was performed on the same day per set of samples. The reference wheat variety “Evina” was used to calculate a correction factor (Equation 1) to account for between-days variation:

(1) $$CF_{j}={\bar{AX}}_{.}/{\bar{AX}}_{.j}$$

Where *CF_j_* is the correction factor for day *j*, ${\bar{AX}}_{.}$ is the average TOT-AX or WE-AX content of all the reference flour samples measured during the experiment, and ${\bar{AX}}_{.j}$ is the average TOT-AX or WE-AX content of the reference flour samples on day *j*. The TOT-AX and WE-AX content of the rye flour samples measured on each *j^th^* day were multiplied by the relevant *CF_j_* and expressed as % of dry matter (dm). *CF_j_* applied to TOT-AX values ranged between 0.93–1.13, and *CF_j_* applied to WE-AX values ranged between 0.94–1.07.

The reagent used for the colorimetric essay consisted of 836 ml glacial acetic acid (VWR Chemicals, Leuven, Belgium), 17.48 ml 37% HCl (Thermo Scientific Chemicals, Brussels, Belgium), 38 ml 10% w/v phloroglucinol (>99.0%; Merck Life Science BV, Hoeilaart, Belgium) in ethanol absolute (VWR Chemicals, Leuven, Belgium), and 7.6 ml 1.75% w/v glucose (99+%, anhydrous; Thermo Scientific Chemicals, Brussels, Belgium) in demineralised water. For the determination of TOT-AX content, 15 mg of flour was weighed directly in a tempered glass tube and mixed with 30 ml of reaction reagent. For the determination of WE-AX content 60 mg of flour was first weighed in a single-use 15 ml falcon tube and mixed with 10 ml demineralized water to obtain a flour aqueous extract by shaking on a SSL1 orbital shaker (Stuart; Keison products, Chelmsford, United Kingdom), for 25 minutes at 150 rpm at room temperature. After centrifugation for 10 minutes at 1,000 × g at room temperature to precipitate the larger flour particles, 2 ml of the flour aqueous extract was transferred to a tempered glass tube and mixed with 10 ml of the reaction reagent. The reaction conditions were the same for the determination of TOT-AX and WE-AX contents and followed Kiszonas et al. (2012), with modifications. Namely, the reaction product was transferred to ROTILABO^®^ single-use cuvettes (Carl Roth, Karlsruhe, Germany) and the absorbance at a wavelength of 553 nm was read using a SPECTROstar Nano spectrophotometer (BMG Labtech, Ortenberg, Germany). The TOT-AX and WE-AX content values of the flour samples were determined based on a standard absorbance response curve. D-xylose (Merck Life Science BV) solutions (i.e., 0.0, 0.05, 0.10, 0.15, 0.20, 0.25, and 0.30 mg/ml) were used. The proportion of WE-AX to TOT-AX (WE/TOT-AX) was calculated as the ratio between WE-AX content and TOT-AX content, and the water-unextractable arabinoxylan (WU-AX) content was calculated as the difference between TOT-AX and WE-AX content, respectively.

### 2.3 Statistical analysis and data visualization

All data visualisation and statistical analysis were performed using R (version 4.3.1; R Core Team, 2023). All graphical output was produced with custom functions built using the libraries *ggplot2, lemon*, *gridExtra*, *scales* (Auguie and Antonov, 2017; Edwards et al., 2024; Wickham et al., 2023, 2024).

Summary statistics were calculated for each phenotype and included maximum, minimum, mean, standard deviation, and coefficient of variation. Kendall’s τ rank correlation coefficient between phenotypes was calculated using the *Kendall* library (Kendall, 1938; McLeod, 2022). A mixed-effect linear model fitted with the *lme4* library (Bates et al., 2024) was used to derive genotypic trait values as best linear unbiased estimates (BLUE). The model (Equation 2) was specified according to Piepho and Williams (2016):

(2) $$Y_{ijklm}=\mu +G_{i}+T_{j}+GT_{ij}+TB_{jk}+TR_{jl}+TC_{jm}+\epsilon_{ijklm}$$

Where *Y_ijklm_* is the phenotypic value of the *i^th^* genotype in the *j^th^* trial, *k^th^* block, *l^th^* super-row, and *m^th^* super-column, *µ* is the grand mean, *G_i_* and *T_j_*, are the main effects of the *i^th^* genotype and of the *j^th^* trial, respectively, *GT_ij_* is the interaction between *i^th^* genotype and *j^th^* trial, *TB_jk_* is the interaction between the *j^th^* trial and the *k^th^* block, *TR_jl_* is the interaction between the *j^th^* trial and the *l^th^* super-row, *TC_jm_* is the interaction between the *j^th^* trial and the *m^th^* super-column, and *ϵ_ijklm_* is the residual. The main effects *G_i_* and *T_j_* were fitted as fixed effects, whereas the interaction terms were fitted as random effects. The optimal model specification for each phenotype was chosen by iteratively removing the random effects until the model with the lowest AIC (Akaike information criterion; Akaike, 1987) value was found. The conditional and marginal R² of each MLM were calculated using the *MuMIn* library (Bartoń, 2023). The significance of the main effects *G_i_* and *T_j_* was tested using a type III Wald F-test with Kenward-Roger estimation of degrees of freedom (Kenward and Roger, 1997) as implemented in the *car* library (Fox et al., 2024). To test for differences between phenotypic means of groups of accessions of different geographical origin, the same MLMs were fitted again with the addition of the interaction between trial and geographical region of origin of the *Secale* accessions as fixed effect. Pairwise comparisons between regional groups were performed using the library *emmeans* (Lenth et al., 2024). *P-*values were adjusted for multiple comparisons using the Benjamini-Hochberg false discovery rate (FDR) method (α = 0.05).

*H²* of TOT-AX, WE-AX, WU-AX, WE/TOT-AX, and TKW was estimated using the best linear unbiased prediction (BLUP)-based definition by Cullis et al. (2006) (Equation 3) as implemented in the library *inti* (Lozano-Isla, 2024):

(3) $$H_{Cullis}^{2}=1-\frac{{\bar{v}}_{\Delta \cdot \cdot }^{BLUP}}{2\sigma_{g}^{2}}$$

Where ${\bar{v}}_{\Delta \cdot \cdot }^{BLUP}$ is the average prediction error variance of all pairwise differences between BLUPs of the genotype effects, and $\sigma_{g}^{2}$ is the genetic variance component.

### 2.4 Genotyping-by-sequencing of the *Secale* collection and SNP panel preparation

Leaves were sampled from one to seven individual plants per accession of the *Secale* collection depending on the availability on the field in 2020–2021 and lyophilised to be stored until use. The lyophilised leaves were cut with scissors in equally sized pieces to collect 20 mg of leaf material for DNA extraction. The DNA was extracted using a KingFisher Flex Purification System following manufacturer’s instructions (Fisher Scientific, Merelbeke, Belgium). Digestion of the extracted DNA (≥ 20 ng/µl) with enzymes *Pst*I*-MspA1*I, library preparation, and sequencing (target average number of reads per library = 4M) were performed by LGC Genomics (Berlin, Germany).

The obtained sequencing data was pre-processed using the *GBprocesS* pipeline (Schaumont, 2020). Briefly, the processing steps performed with *GBprocesS* included demultiplexing, trimming of barcodes, remnants of restriction sites and adapter sequences, merging of forward and reverse reads, base call quality filtering of reads, and the removal of reads with intact internal restriction sites. The reads were mapped to the rye “Lo7” genome assembly (Rabanus-Wallace et al., 2021) using the BWA-MEM algorithm (Li, 2013). SMAP *delineate* was used to identify high-quality GBS loci that were consistently called across the sample set (Schaumont et al., 2022). SNAPE-pooled was used to call SNPs per sample within the GBS loci identified (Raineri et al., 2012). The minimum read depth required for SNP calling was set to 30. SNP calls were filtered based on call-rate ≥ 0.9, number of samples with two alleles ≥ 5, and reference allele frequency (RAF) between 0.05 and 0.95. The obtained SNP panel was prepared for the downstream population structure analyses and GWAS with a custom R script (version 4.3.1; R Core Team, 2023). Imputation of missing data was done by *k*-nearest neighbour algorithm as implemented in the *VIM* package, with default settings (Kowarik and Templ, 2016).

### 2.5 Population structure analysis

Discriminant analysis of principal components (DAPC) as implemented in the *adegenet* library (Jombart and Ahmed, 2011) was used to perform a population structure analysis of the *Secale* accessions included in the GBS experiment. The optimal number of clusters *K* was determined by running successive *K-*means clustering with an increasing number of clusters *K* (i.e., 1–10), and by choosing the value of *K* which minimises the Bayesian information criterion (BIC) value. The number of principal components (PCs) to retain during the optimisation of *K* was equal to the number of PCs preserving 90% of total variance. The number of PCs to retain in the PCA step of the DAPC was set to *K* - 1 (Thia, 2023). The number of discriminant functions retained was equal to three (i.e., the maximum).

### 2.6 Genome-wide association study and identification of candidate genes

The GWAS was performed with a custom R script (version 4.3.1; R Core Team, 2023). A Bayesian-information and Linkage-disequilibrium Iteratively Nested Keyway (BLINK) procedure, as implemented in the GAPIT package, was used to test for association between SNP markers and phenotypic traits (Huang et al., 2019). Possible deviations in the obtained *p-*values were visually assessed by means of Q-Q plots. Only SNPs with *p-*value< 0.05 after Bonferroni correction for multiple testing were reported as MTAs.

Candidate genes were searched within the range of linkage disequilibrium (LD) decay around the MTAs output of the GWAS. LD was calculated per chromosome as the squared Pearson’s correlation coefficient (*r²*) between pairs of markers within windows of 10 Mbp. A Locally Estimated Scatterplot Smoothing (LOESS) curve was fitted to the calculated LD distributions using the *stats* library and the distance at which intrachromosomal LD decays was calculated as the intersection between the LOESS curve and the critical *r²*, equal to the 95^th^ percentile (Breseghello and Sorrells, 2006). This physical distance in base pairs (bp) was used to define the upstream and downstream boundaries around the MTAs output of the GWAS. The average LD decay distance was used to define upstream and downstream boundaries for chrUn (i.e., the pseudomolecule comprising concatenated unanchored scaffolds). The candidate genes with cell wall and grain filling-related functions were selected using the gene feature annotation of the rye “Lo7” reference, which was retrieved from e!DAL (Lux and Helmzholtz Zentrum München, 2020).

## 3 Results

### 3.1 Variation of grain quality parameters of rye

The number of *Secale* accessions phenotyped in trial S20/H21 was 267, and 235 in trial S21/H22. The number of *Secale* accessions that was phenotyped in trial S20/H21 as well as in trial S21/H22 was 208. The harvest of trial S20/H21 was characterised by higher WE-AX content and WE/TOT-AX, but lower TKW compared to trial S21/H22 (**Table 1**, **Supplementary Table 2**).

**Table 1:** Summary statistics per phenotype per trial, and summary statistics of the genotypic trait values as best linear unbiased estimates (BLUE).

Data set	Summary statistics	TOT-AX (% dm^a^)	WE-AX (% dm^a^)	WU-AX (% dm^a^)	WE/TOT-AX	TKW (g; 15% gm^b^)
S20/H21 (N = 267)	Maximum	4.63	2.16	2.66	0.65	65.17
	Minimum	2.18	0.95	0.75	0.29	20.20
	Mean ± sd	3.15 ± 0.38	1.48 ± 0.18	1.67 ± 0.28	0.47 ± 0.04	38.10 ± 7.95
	CV	12.10%	12.42%	16.60%	8.99%	20.87%
S21/H22 (N = 235)	Maximum	5.27	2.04	4.10	0.68	71.34
	Minimum	2.41	0.94	0.88	0.22	23.58
	Mean ± sd	3.19 ± 0.51	1.31 ± 0.17	1.88 ± 0.46	0.42 ± 0.06	44.57 ± 8.39
	CV	15.97%	12.93%	24.45%	14.05%	18.82%
BLUE (N = 208)	Maximum	4.63	2.04	2.98	0.60	62.52
	Minimum	2.49	1.19	1.08	0.34	20.71
	Mean ± sd	3.19 ± 0.39	1.49 ± 0.14	1.70 ± 0.32	0.47 ± 0.04	38.06 ± 7.77
	CV	12.27%	9.41%	18.87%	8.64%	20.41%

S20/H21, field trial of growing season 2020-2021; S21/H22, field trial of growing season 2021-2022; TOT-AX, total arabinoxylan content; WE-AX, water-extractable arabinoxylan content; WU-AX, water-unextractable arabinoxylan content; WE/TOT-AX, proportion of WE-AX to TOT-AX; TKW, thousand kernel weight, sd: standard deviation; CV, coefficient of variation.

^a^Expressed as % of dry matter (dm).

^b^Normalised to 15.0% grain moisture (gm).

**Figure 2:**
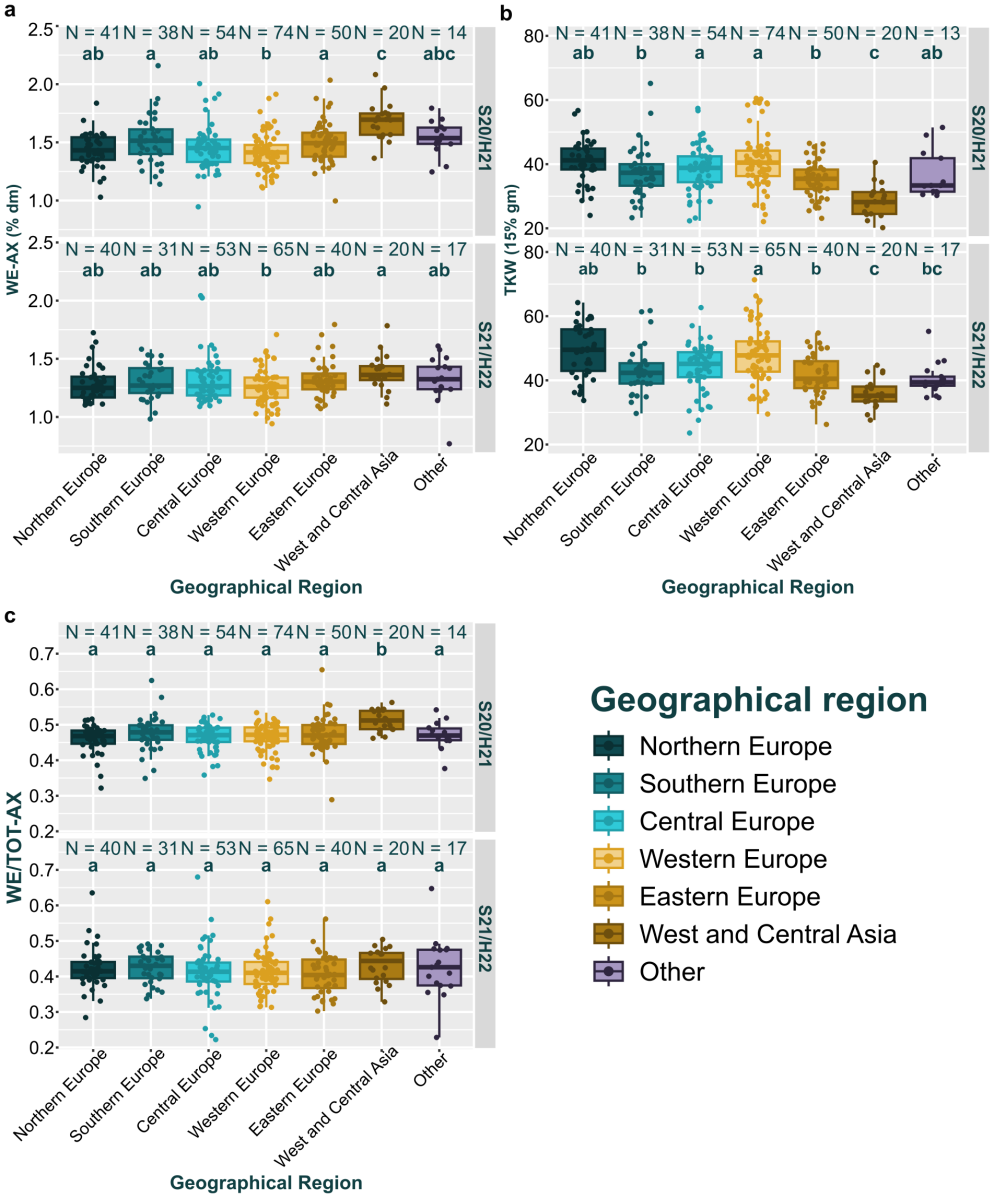
Boxplot distributions of water-extractable arabinoxylan content [**(a)**, WE-AX % dry matter (dm)], thousand kernel weight [**(b)**, TKW g normalised to 15% grain moisture (gm)], and the proportion of water-extractable arabinoxylan to total arabinoxylan content [**(c)**, WE/TOT-AX] stratified by trial (S20/H21: trial of season 2020-2021, S21/H22: trial of season 2021-2022) and by geographical region of origin of the accessions (**Supplementary Table 1**). The number of accessions per group (N) is noted above each box. Within one trial, different letters denote a statistically significant difference at α = 0.05.

In trial S20/H21 there was a significant difference of WE-AX content and WE/TOT-AX between the group of accessions from West and Central Asia and all other groups, whereas in trial S21/H22 there was a statistically significant difference only of WE-AX content, and only between the West and Central Asia group and the Western Europe group (**Figures 2a, b**). Statistically significant differences in TKW between regional groups within both trials were also observed (**Figure 2c**). In particular, TKW tended to be higher for the accessions from Western and Northern Europe, whereas the lowest TKW was obtained for the accessions from West and Central Asia group. No statistically significant difference of TOT-AX and WU-AX contents between geographical groups of accessions was observed in both trials (**Supplementary Figure 1**).

TOT-AX content was negatively correlated with WE/TOT-AX (τ = -0.246) and TKW (τ = -0.166), but positively correlated with WE-AX content (τ = 0.361) and WU-AX content (τ = 0.679). WE-AX content was negatively correlated with TKW (τ = -0.300), positively correlated with WE/TOT-AX (τ = 0.393) and uncorrelated with WU-AX content (τ = 0.040). WU-AX was negatively correlated with WE/TOT-AX (τ = -0.567) and uncorrelated with TKW (τ = -0.007). WE/TOT-AX was negatively correlated with TKW (τ = -0.215).

Genotype was a significant predictor for all traits (p-value< 0.001), whereas trial was a significant predictor for all traits except TOT-AX content. A moderate *H²* was estimated for TOT-AX and WU-AX contents (*H²*_TOT-AX_ = 0.65, *H²*_WU-AX_ = 0.53), whereas a low *H²* was estimated for WE-AX content and WE/TOT-AX (*H²*_WE-AX_ = 0.33, *H²*_WE/TOT-AX_ = 0.29). A high *H²* was estimated for TKW (*H²*_TKW_ = 0.87; **Supplementary Table 3**).

### 3.2 Genotyping-by-sequencing of the *Secale* collection

**Figure 3:**
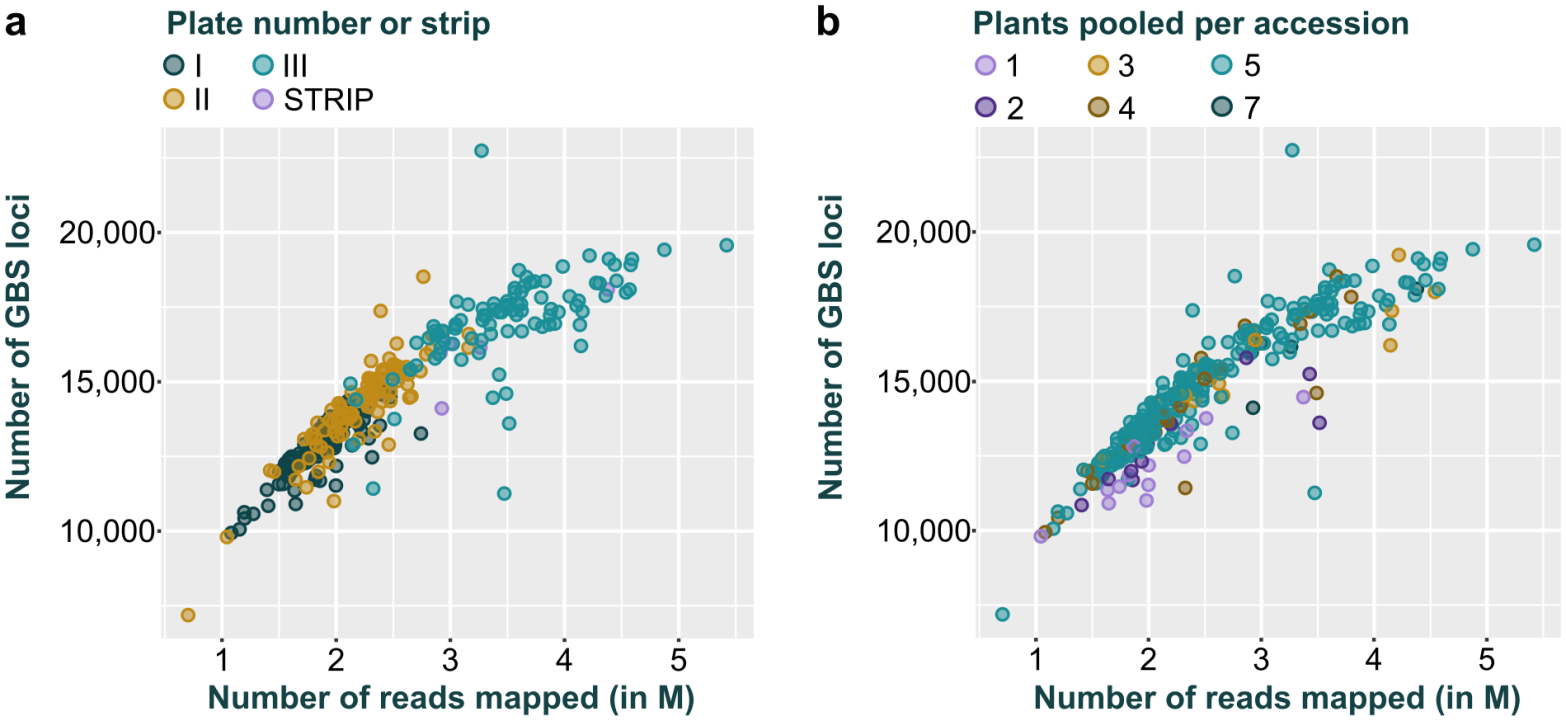
Saturation curves of the genotyping-by-sequencing (GBS) experiment on the *Secale* collection stratified by 96-well plate or strip number **(a)**, and by number of plants pooled per accession **(b)**.

A total of 294 genotypes were included in the GBS experiment on the *Secale* collection, including 289 of the 312 *Secale* accessions and all five rye check varieties. Two-thirds of the samples included in the GBS experiment were not sequenced to saturation (**Figure 3a**). Stratification of the saturation curve by number of plants pooled per accession or by sample position on a certain 96-well plate or strip showed that the number of plants pooled per accession did not affect the sample saturation, but rather, that there was a correlation between sample saturation and sample position on a certain 96-well plate or strip (**Figures 3a, b**).

A total of 37,907 unique GBS loci were obtained. A total of 49,498 SNPs were called from 5,976 of the obtained GBS loci (10.5281/zenodo.21064232). The average distance between GBS loci containing SNPs was 1.1 Mbp. SNP coverage was comparable between the seven chromosomes, with a range of 3–4 SNPs per 100 bp of genome covered by GBS loci with call-rate ≥ 0.9 (**Table 2**).

**Table 2:** Overview of the SNP coverage for each of the seven chromosomes of rye (chr1R, chr2R, chr3R, chr4R; chr5R, chr6R, chr7R) and for the concatenated unanchored scaffolds (chrUn).

Chromosome	Chromosome length (bp)	Length (bp) covered by GBS loci with call-rate ≥ 0.9	Fraction covered with call-rate ≥ 0.9 (%)	Total number of SNPs per chromosome	Number of SNP per 100 bp of covered genome with call-rate ≥ 0.9
chr1R	727,197,289	156,109	0.021	6,487	4.2
chr2R	945,846,888	219,451	0.023	7,702	3.5
chr3R	965,480,912	175,600	0.018	6,000	3.4
chr4R	906,382,516	196,706	0.022	7,831	4.0
chr5R	876,053,702	192,280	0.022	7,233	3.8
chr6R	884,803,034	197,246	0.022	6,921	3.5
chr7R	899,765,510	175,352	0.019	5,846	3.3
chrUn	526,947,010	37,016	0.007	1,478	4.0

### 3.3 Population structure analysis

**Figure 4:**
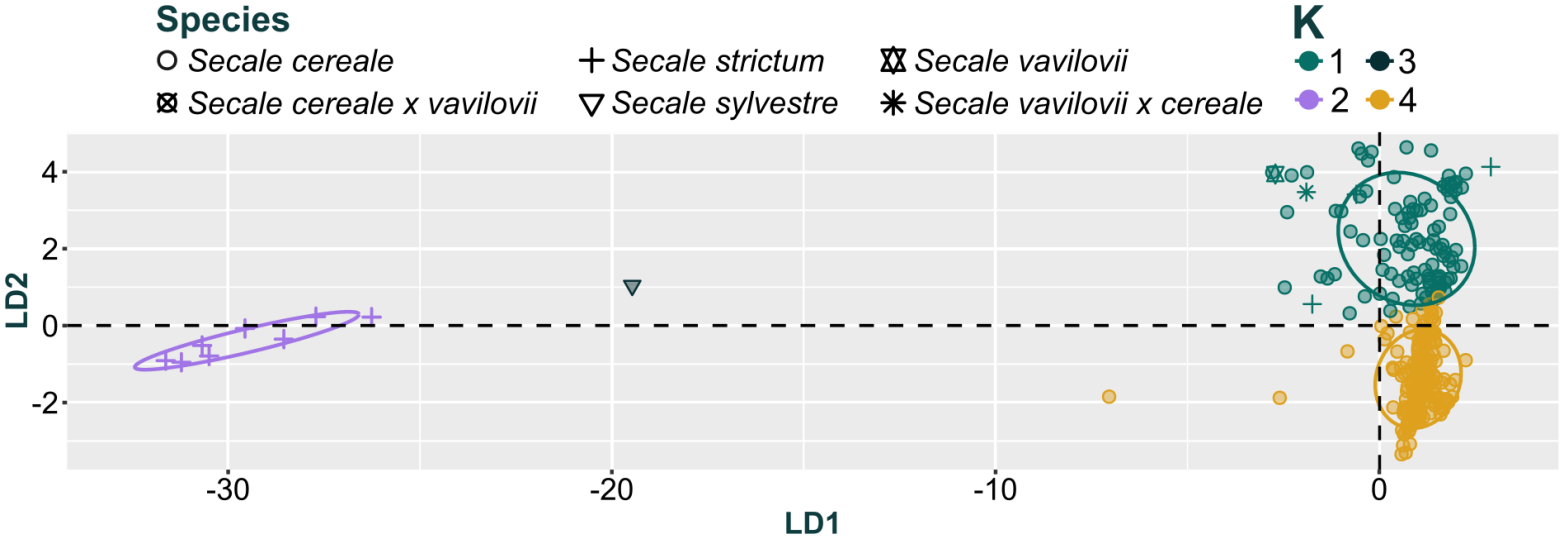
Biplot of the discriminant analysis of principal components (DAPC) of the *Secale* collection. Axes represent the first and the second linear discriminants (LD1, LD2). Each point represents an individual accession. Points are coloured based on their assignment to a specific subpopulation cluster (K). Different shapes indicate the species of each accession (○: *S. cereale*, ⊗: *S. cereale* × *S. vavilovii*, +: *S. strictum*, ▽: *S. sylvestre*, ✡: *S. vavilovii*, ✳: *S. vavilovii* × *S. cereale*).

Successive *k*-means clustering resulted in *k* = 4 as the optimal number of clusters in which to subdivide the collection (**Figure 3**, **Supplementary Figure 2**, **Supplementary Table 4**). The clusters well divided the accessions based on species, so that *S. strictum* and *S. sylvestre* accessions formed their own clusters. However, three of the 11 *S. strictum* accessions clustered with *S. cereale* accessions. *S. vavilovii* and hybrid accessions (i.e., *S. cereale* × *S. vavilovii* and *S. vavilovii* × *S. cereale*) also clustered together with *S. cereale* accessions. *S. cereale* accessions were divided into two different clusters (**Figure 4**).

**Figure 5:**
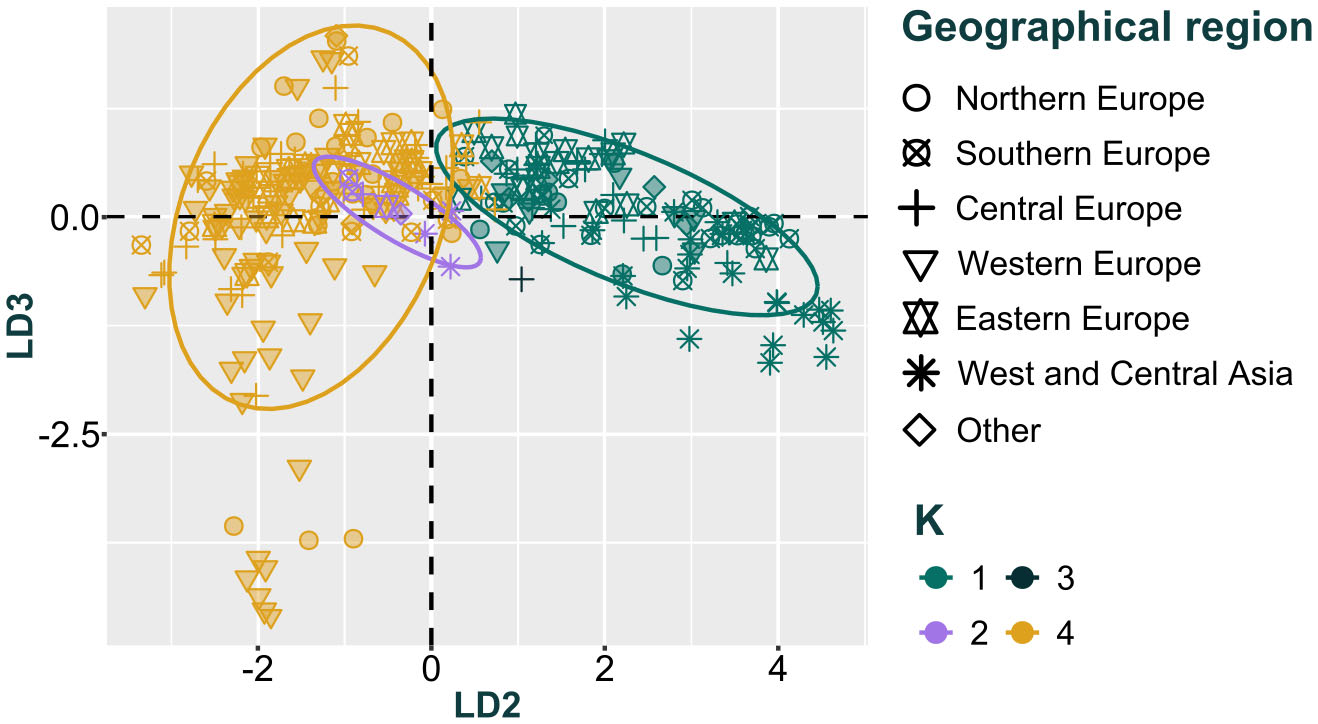
Biplot of the discriminant analysis of principal components (DAPC) of the *Secale* collection. Axes represent the second and the third linear discriminants (LD2, LD3). Each point represents an individual accession. Points are coloured based on their assignment to a specific subpopulation cluster (K). Different shapes indicate the geographical region of origin of each accession (○: Northern Europe, ⊗: Southern Europe, +: Central Europe, ▽: Western Europe, ✡ Eastern Europe, ✳: West and Central Asia, ◇: Other).

The dispersion of the accessions across the space defined by the second and third linear discriminants showed that the two clusters comprising *S. cereale* accessions separate these accessions according to their geographical origin (**Figure 5**).

Accessions originating from West and Central Asia and those originating from Southern Europe tended to be grouped in the same cluster (i.e., K1). The other cluster comprising *S. cereale* accessions (i.e., K4) tended to be mostly comprised of accessions originating from Western Europe, Central Europe, and Northern Europe (**Supplementary Table 1**). Accessions from Eastern Europe were spread comparably across these two clusters (**Figure 5**).

### 3.4 Genome-wide association study of grain quality parameters

**Figure 6:**
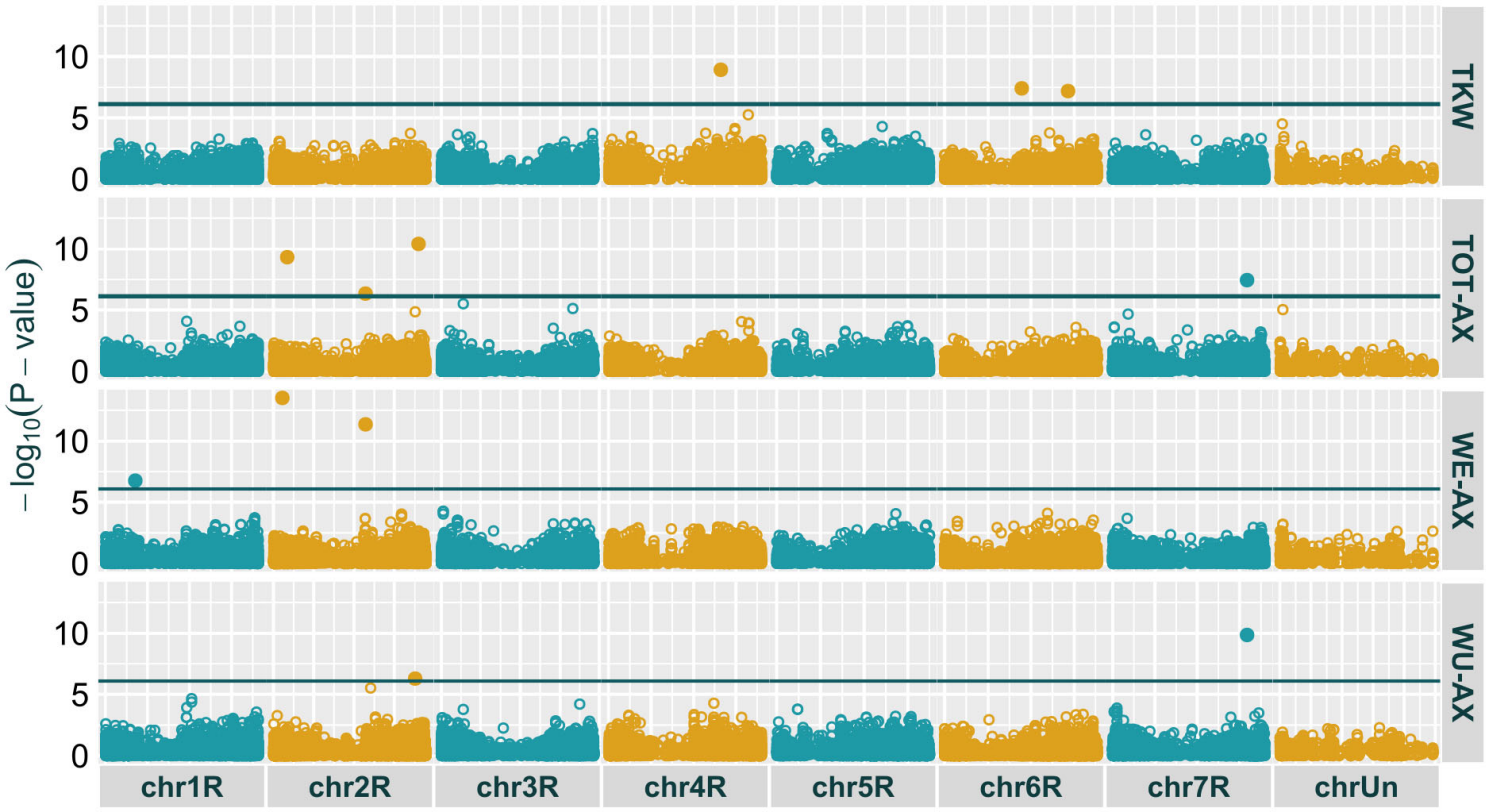
Manhattan plots showing the results of the GWAS for grain quality traits of rye. The abscissa represents the chromosomal coordinates of each tested SNP, whereas the ordinate represents the -*log*(*p*-value) per SNP as obtained through the BLINK procedure. The solid horizontal line represents the significance threshold α = 0.05 after Bonferroni correction. TOT-AX: total arabinoxylan content, WE-AX: water-extractable arabinoxylan content, WU-AX: water-unextractable arabinoxylan content, TKW: thousand kernel weight.

The GWAS resulted in 12 statistically significant MTAs (**Figure 6**, **Supplementary Figures 3**, **4**, **Table 3**). Four MTAs were associated to TOT-AX content, three to WE-AX content, two to WU-AX content, and three to TKW. No MTA was detected for WE/TOT-AX.

**Table 3:** List of statistically significant MTAs (*p-*value< 0.05 after Bonferroni correction) for total arabinoxylan (TOT-AX), water-extractable arabinoxylan (WE-AX), water-unextractable arabinoxylan (WU-AX) contents, and thousand kernel weight (TKW).

Associated trait	SNP	Chromosome	Position (bp)	*p*-value	PVE (%)
TOT-AX	chr2R_85169289	2R	85,169,289	4.88×10^-10^	4.33
TOT-AX	chr2R_570534166	2R	570,534,166	4.55×10^-7^	0.80
TOT-AX	chr2R_898983225	2R	898,983,225	4.04×10^-11^	0.75
TOT-AX	chr7R_792999855	7R	792,999,855	3.64×10^-8^	30.72
WE-AX	chr1R_140892057	1R	140,892,057	1.74×10^-7^	5.44
WE-AX	chr2R_54768648	2R	54,768,648	3.09×10^-14^	17.90
WE-AX	chr2R_570534254	2R	570,534,254	4.44×10^-12^	21.43
WU-AX	chr2R_878199355	2R	878,199,355	5.14×10^-7^	26.08
WU-AX	chr7R_792999855	7R	792,999,855	1.42×10^-10^	32.56
TKW	chr4R_661716055	4R	661,716,055	1.19×10^-9^	13.73
TKW	chr6R_446990889	6R	446,990,889	3.39×10^-8^	7.98
TKW	chr6R_715671769	6R	715,671,769	6.52×10^-8^	33.13

PVE, phenotypic variance explained. SNPs are coded based on chromosome and position in base pairs (bp).

The average LD decay distance was 277 Kbp (**Supplementary Table 5**). A total of 53 genes were annotated within the LD decay range around each MTA (**Supplementary Table 6**). Considering the MTAs for TOT-AX, WE-AX, and WU-AX contents, only one of the candidate genes, SECCE2Rv1G007557, was annotated with a cell wall-related function. Gene SECCE2Rv1G007557 is located on the short arm of chromosome 2R, within LD decay range of SNP chr2R_85169289, and it is associated with TOT-AX content. This gene encodes an endo-1,4-beta-xylanase from the glycosyl hydrolase family 10 (GH10). Notably, only one gene (SECCE7Rv1G0510700) was found within LD range of SNP chr7_792999855, which constitutes a major MTA for both TOT-AX (PVE = 30.72%) and WU-AX (PVE = 32.56%). This gene encodes a RNA-binding (RRM/RBD/RNP motifs) family protein.

Considering the MTAs for TKW, no candidate genes related to the biosynthesis of storage proteins or starch were found within LD decay range. Six of the candidate genes within LD range of the MTAs for TKW were annotated with cell-wall related functions, including hydroxycinnamoyl-transferase, uridine 5’-diphosphate (UDP) glycosyltransferase and Casperian strip membrane protein (CASP)-like protein.

## 4 Discussion

### 4.1 Environmental conditions and selection for a specific end use affect grain quality of rye in terms of arabinoxylan contents and thousand kernel weight

A wet and cool grain filling period determines an increase in WE-AX content in wheat (Dornez et al., 2008). Similarly, in this collection of rye, a higher WE-AX content and WE/TOT-AX was obtained in trial S20/H21, which was characterized by a rather rainy grain filling period as compared to trial S21/H22 (**Figure 1**, **Table 1**). Trial S20/H21 was also characterised by a shorter grain filling period and an overall shorter crop growth cycle compared to trial S21/H22. Moreover, the sowing of trial S20/H21 happened only in December 2020, which exposed the young rye plants to cold winter weather early on. All of these conditions together likely concurred in the determination of the lower TKW obtained in trial S20/H21 (**Figure 1**, **Table 1**), since TKW is a complex trait that is affected by the length of the growth cycle, as well as by the environmental conditions before and right after the winter period, and during grain filling (Hansen et al., 2003; Wang et al., 2023).

The end use of rye is closely related to the geographical region where rye is cultivated (Parat et al., 2016), and it has been argued that selection for a specific end use has determined genetic separation among European rye accessions (Parat et al., 2016). This was phenotypically evident in the *Secale* collection. TKW was higher for regional groups where rye is cultivated as a cereal for food, such has Northern and Western Europe, but lower for regional groups where rye is cultivated as a forage, such as Southern Europe and West and Central Asia (**Figure 2c**). The high *H²* estimate for TKW in this *Secale* collection further highlights the presence of these genetically distinct groups. Notably, the geographical origin of the accessions did not influence the AX contents of the rye accessions except for the West and Central Asia group (**Figures 2a, b**, **Supplementary Figure 1**). However, the higher WE-AX contents of this group could be attributed to the low adaptation of these accessions to the environmental conditions during the field trials in Belgium. Overall, these results suggest that there has not been any selection on AX content in rye, irrespective of the intended end use. The moderate to low Cullis-based *H²* estimates for the AX-related traits partly reflect the design of the field experiment, because limited replication of the augmented design increases the prediction error variance of pairwise contrasts between BLUPs of the genotypic effects. However, these estimates also suggest a large impact of the environment on the AX content in this *Secale* collection.

### 4.2 Selection for a certain end use is a driver of genetic diversification between geographical groups of *Secale cereale*

DAPC analysis of the *Secale* collection separated *S. strictum* and *S. cereale* accessions in two very distinct clusters, in agreement with previous analyses of population structure in *Secale* ssp (Hawliczek et al., 2023; Schreiber et al., 2019). Close relatedness between *S. vavilovii* and *S. cereale* is also well documented (Hawliczek et al., 2023; Schreiber et al., 2019). Notably, two accessions known in the collection as *S. strictum* clustered together with *S. cereale* accessions (**Figure 4**). This suggests inaccurate maintenance or labelling of this material. The separation of *S. cereale* accessions into two clusters defined two rather uniform groups in terms of geographical origin of the accessions. These two groups separate geographical regions where rye is grown for its grain, from countries where rye is mostly grown as forage, supporting that selection for a determinate end use has driven genetic diversification of the rye germplasm (Korzun et al., 2021; Parat et al., 2016). Notably, accessions from Eastern Europe were equally abundant between these groups. This is possibly because rye is exploited for both end uses in this region (Korzun et al., 2021).

### 4.3 Genome-wide association study of arabinoxylan contents in rye points to known genomic locations but candidate genes remain elusive

Boros et al. (2002), identified several chromosomes of rye, including 2R, 5R, 6R, 3RL, and 7R, as responsible for the determination of DF and AX content using a variety of wheat-rye disomic addition and translocation lines. The research of Szakács et al. (2016, 2020), further suggested that chromosomes 1R and 4R also play a role in the determination of AX content in rye. This work appears to partially validate their results (**Table 3**). Six MTAs for TOT-AX, WE-AX, and WU-AX contents were found spread across chromosome 2R, supporting the hypothesis that this chromosome plays a crucial role in the regulation of AX content in the rye grains. Boros et al. (2002), also suggested that antagonistic loci were present on the short and long arm of chromosome 7R. While an MTA with major effects on WU-AX and TOT-AX contents was found on the long arm of chromosome 7R, no MTA was found on the short arm of this chromosome (**Table 3**). The average LD decay range in this *Secale* collection was smaller than the average distance between two GBS loci containing SNPs. Furthermore, if we consider the range of LD decay around all SNPs located on chromosomes 3R, 5R, and 6R (**Table 2**, **Supplementary Table 5**) then this study could effectively survey 30% of the total length of chromosome 3R, 61% of chromosome 5R, and 65% of chromosome 6R. Thus, the relatively low density of polymorphic GBS loci might have precluded the detection of MTAs on chromosomes 3RL, 5R, and 6R.

Despite the large effects of several of the MTAs, candidate genes for AX contents in rye remain elusive, with only one candidate gene annotated with a cell wall-related function found in this study. Gene SECCE2Rv1G007557 encodes an endo-1,4-beta-xylanase from the GH10 family that is orthologous to the maize (*Zea mays* L.) gene *Zmxyl1* which remodels the cell wall by degrading xylan (Hu et al., 2020). This gene was previously found to be continuously expressed in rye kernels throughout development, with expression being the highest at milk stage (Kozlova et al., 2022). AX is deposited very early during grain development of wheat and is continuously restructured during development and in response to the environment (Toole et al., 2007, 2009). The same dynamic deposition and restructuring could be expected for rye AX. Thus, cell wall reorganisation appears to be a determinant factor affecting the TOT-AX content in the mature rye kernels.

### 4.4 The regulation of nutrient and hormone fluxes may affect thousand kernel weight in rye

Candidate genes related to the restructuring of the cell wall and the regulation of grain size were also found within LD range of SNPs associated with TKW. The most interesting candidates are gene SECCE6Rv1G0426790, encoding a CASP-like protein, and gene SECCE6Rv1G0426720 encoding an UDP-glycosyltransferase. Although CASP-like proteins are proteins related to the deposition of the Casperian strip in the endodermis of roots (Barbosa et al., 2023; Roppolo et al., 2014), a CASP-like protein-encoding gene was found expressed in the endosperm and embryo of sorghum (*Sorghum bicolor* Moench) and associated with starch content (Sapkota et al., 2020). Possibly, these CASP-like proteins participate in the regulation of nutrient transfer from the mother plant to the seed (Sapkota et al., 2020). UDP-glycosyltransferase catalyse the glycosylation of flavonoids, which are important regulators of auxin transport and therefore the regulation of seed size (Dong et al., 2020; Liu et al., 2015). In rice (*Oryza sativa* L.) mutants lacking active UDP-glycosyltransferase exhibited accumulated aglycone flavonoids, leading to an impaired auxin transport and significantly reduced grain size (Dong et al., 2020). Possibly, this mechanism affecting grain size is shared across the members of the family of the *Poaceae*, including rye.

### 4.5 The introgression of chromosome segments 2RL and 7RL could increase AX content in wheat flour, but substantial effects on agronomic performance should be expected

The introgression of a chromosome segment entails the introgression of several genes, affecting a variety of traits. The study on the *Secale* collection has shown a negative correlation between TOT-AX or WE-AX content and TKW. Considering that WU-AX content was not correlated with TKW, it can be argued that WE-AX is the driver of this negative correlation (Section 3.1). This result suggests a possible genetic relationship between TOT-AX, WE-AX and TKW. The MTAs for TOT-AX and WE-AX contents that were detected in this study were spread across chromosomes 1R, 2R, and 7R (**Table 3**). None of these MTAs colocalised with the MTAs for TKW (**Figure 6**), which were detected on chromosomes 4R and 6R. However, Hackauf et al. (2017), have mapped on the long arm of chromosome 2R a QTL for TKW orthologous to a QTL identified in rice. Possibly, in the *Secale* collection, the distance between this QTL and the nearest polymorphic GBS locus is greater than the LD decay distance on chromosome 2R. Therefore, further attention should be dedicated to the elucidation of the genetic relationship between the determination of TKW and of WE-AX content in the rye grain.

Chromosome 1R and chromosome segment 2RS are not interesting as rye introgression targets in bread wheat because of the *Sec-1*, *Sec-2*, *Sec-3*, and *Sec-4* loci encoding secalin storage proteins that are located in these regions (Shewry et al., 1986; Li et al., 2021). The introgression of secalin loci in bread wheat has been shown to be detrimental to gluten quality (Graybosch, 2001). To our knowledge, no known loci encoding secalins are located on the long arms of chromosome 2R and 7R (Li et al., 2021). These chromosomal segments remain as new candidate introgression targets for the improvement of AX content in wheat flour. Besides the aforementioned QTL for TKW, the self-incompatibility locus *Z* and several QTLs for agronomic traits including grain yield, plant height, and heading date have been mapped to segment 2RL (Hackauf et al., 2017; Hackauf and Wehling, 2005; Plaschke et al., 1995). Therefore, the consequences on the agronomic performance of 2RL introgression lines should be thoroughly evaluated. The gibberellin-insensitive dwarfing locus *ct1* is thought to span around the centromere of chromosome 7R, and to be in genetic linkage to α-Amy2 genes (Masojć and Gale, 1991; Plaschke et al., 1995). The rye grain is characterized by a high α-amylase activity, which can cause excessive starch degradation, thus leading to soft, sticky doughs (Deleu et al., 2020). Furthermore, a high α-amylase activity is associated with pre-harvest sprouting (Hull et al., 2024). Selection of rye 7RL donors based on the α-amylase activity would be required to avoid detrimental effects to wheat grain and dough quality.

## 5 Conclusion

The end use typical in a certain geographical region has determined the genetic diversification of the rye gene pool across Europe and West and Central Asia. This genetic diversification is phenotypically evident when considering TKW of rye but not when considering AX content in flour. The GWAS identified four MTAs for TOT-AX, three for WE-AX, two WU-AX content, and three for TKW, for a total of 12 MTAs. Despite the lack of candidate genes with known cell wall-related functions within LD range of the associated SNPs, given the generally high proportion of phenotypic variance explained, these MTAs deserve further attention as introgression targets for AX-targeted breeding in wheat, particularly the MTAs located on the long arms of chromosome 2R and 7R. Three MTAs were also found for TKW. These associations point to intriguing regulatory mechanisms of the flux of nutrients and hormones towards the developing seed.

## Data Availability

The datasets generated and used to perform the GWAS presented in this article can be found at the Zenodo online repository: 10.5281/zenodo.21064232.
