# Supplementary material for: Identification of targets for introgression from rye (Secale ssp.) into wheat (Triticum aestivum) to increase arabinoxylan content in flour using a genome-wide association study
Source: Front Plant Sci. 2026 Jul 22;17:1892461. doi: 10.3389/fpls.2026.1892461 (PMC13437688; doi:10.3389/fpls.2026.1892461)
Supplement: Supplementary file 1 [file DataSheet1.docx]

Supplementary Material

Supplementary Figure 1. Boxplot distribution of total (TOT-AX) and water-unextractable arabonoxylan (WU-AX) contents in rye flour (% dry matter [dm]) stratified by trial (S20/H21: 2020-2021, S21/22: 2021-2022) and by geographical region of origin of the accessions (Supplementary table 1). The number of accessions (N) per group is noted above each box. Within one trial, different letters denote a statistically significant difference at α = 0.05).

**Supplementary Figure 2.** Plot of BIC value versus number of clusters.

**Supplementary Figure 3.** QQ-plot of the observed versus expected distribution of the -*log*(*p*-values) obtained from the GWAS for grain quality traits of rye. Different colours indicate the distribution of *p*-values obtained when testing against a different phenotype. TOT-AX: total arabinoxylan content (%, dry matter [dm]), WE-AX water-extractable arabinoxylan content (% dm), WU-AX: water-unextractable arabinoxylan content (% dm), WE/TOT-AX: proportion of WE-AX to TOT-AX, TKW: thousand kernel weight (g 15% grain moisture).

**Supplementary Figure 4.** Manhattan plot output of the genome-wide association study for the proportion of total arabinoxylan content to water-extractable arabinoxylan content (WE/TO-AX) in rye flour.
